# Accurate and efficient protocols for high-throughput first-principles materials simulations

**DOI:** 10.1038/s41524-026-02097-8

**Published:** 2026-04-29

**Authors:** Gabriel de Miranda Nascimento, Flaviano José dos Santos, Marnik Bercx, Davide Grassano, Giovanni Pizzi, Nicola Marzari

**Affiliations:** 1 Theory and Simulation of Materials (THEOS), École Polytechnique Fédérale de Lausanne, Lausanne, Switzerland; 2 PSI Center for Scientific Computing, Theory and Data, Villigen PSI, Switzerland; 3 National Centre for Computational Design and Discovery of Novel Materials (MARVEL), Paul Scherrer Institute PSI, Villigen PSI, Switzerland; 4 Brazilian Center For Research in Physics (CBPF), Rio de Janeiro, Brazil

**Keywords:** Electronic structure, Computational methods

## Abstract

Advancements in theoretical and algorithmic approaches, workflow engines, and an ever-increasing computational power have enabled a novel paradigm for materials discovery through first-principles high-throughput simulations. A major challenge in these efforts is to automate the selection of parameters used by simulation codes to deliver numerical precision and computational efficiency. Here, we propose a rigorous methodology to assess the quality of self-consistent DFT calculations with respect to smearing and *k*-point sampling across a wide range of crystalline materials. For this goal, we develop criteria to reliably estimate average errors on total energies, forces, and other properties as a function of the desired computational efficiency, while consistently controlling *k*-point sampling errors. The present results provide automated protocols (named standard solid-state protocols or SSSPr) for selecting optimized parameters based on different choices of precision and efficiency tradeoffs. These are available through open-source tools that range from interactive input generators for DFT codes to high-throughput workflows.

## Introduction

Density-functional theory (DFT) has been a very successful approach to address the ground state of many-electron systems and has evolved into a predictive tool for materials discovery and design^1,2^. The predictive power of the theory, the robust implementations provided by DFT codes, and increasing available computational power in the past years have unlocked the potential of high-throughput approaches for materials discovery^3–5^, where automated computational workflows are leveraged for the study and simulation of a large number of compounds. Starting from experimentally known crystal structures, these efforts are responsible for the construction and curation of numerous computational databases of materials^6–10^, where several properties of many known compounds are addressed with various levels of accuracy. Moreover, data for these approaches serve as a starting point for strategies that aim at generating completely novel crystal structures, with approaches that range from random structure searching^11^, structural prototyping^9^ to genetic algorithms^12,13^ and machine-learning-powered generative models^14,15^.

To properly manage these efforts on high-throughput calculations and support their scalability needs, workflow engines have been developed and coevolved with DFT codes. They provide different levels of functionality that are suited for multiple use cases, ranging from input generation/output parsing and managing input/output operations, to interfacing with computational facilities and schedulers. Examples include materials APIs such as pymatgen^16^ and ASE^17^, and workflow managers such as FireWorks^18^, Pyiron^19^, Atomic Simulation Recipes^20^ and AiiDA^21–23^. In such frameworks, one of the main challenges in designing robust and efficient high-throughput workflows is achieving a reliable balance between precision and computational cost. On the one hand, producing consistent and highly converged calculations is not only important for achieving the highest precision of results for a given level of theory, but it is also crucial for the success of many downstream applications. A paramount example is the generation of data for training machine learning models. The importance of having consistent and highly converged data was underscored by Dragoni et al.^24^, and more recently showcased by Mazitov et al.^25^ and Marchand^26^ in the development of universal interatomic potentials and foundation models. On the other hand, large-scale computational workflows also demand a stronger emphasis on computational efficiency, since they involve workloads that may scale to tens of thousands of materials and hundreds of thousands of DFT calculations. Optimizing the methods and inner parameters in these calculations such that they run as efficiently as possible, and ensuring that no computational resource is wasted and not converted into precision gains is, therefore, an essential part of designing robust computational workflows for first-principles materials simulations. Remarkable efforts in this direction include the creation of standard solid-state pseudopotential libraries (SSSP)^27^, that established a testing protocol based on extensive DFT and density-functional perturbation theory (DFPT) calculations using the main pseudopotential libraries available^28–34^. Using these protocols, SSSP curates an exhaustive and open collection of extensively tested pseudopotentials and related parameters, suitable for different use cases of high-throughput and high-precision materials modeling.

This work contributes to solutions to these challenges by introducing the concept of standard solid-state protocols (SSSPr) as an extended framework to the SSSP pseudopotentials^27^. They are defined as a collection of parameters for specific codes and calculations, optimized for different levels of precision and computational efficiency and extensively tested for a wide range of materials. These operate within computational workflows, acting as an interface between their higher-level logic of operations and the inner-level details of parameters for a given calculation called by the workflow. More specifically, in this work we build upon the AiiDA workflow framework and its plugin and workflows for Quantum ESPRESSO^35–38^, a plane-wave pseudopotential DFT code. However, the methods developed here can be applied to any other code/sampling methodology. In these protocols, we start from the results obtained by Prandini et al.^27^ for the standard solid-state pseudopotentials (SSSP) library and extend these to further benchmarks for the additional parameters that most strongly influence the precision and computational cost of DFT calculations for crystals: namely, *k*-point sampling and smearing techniques. For this reason, we define a new acronym for the standard solid-state protocols (SSSPr), paralleling the original SSSP acronym referring to the pseudopotentials (which are, in turn, used in the SSSPr protocols). Similar work in this direction has been most notably performed by Choudhary and Tavazza^39^, where they employed a systematic procedure for benchmarking *k*-point grids and plane-wave energy cutoffs in calculations run with VASP^40,41^, and has also been developed along the construction of materials databases, such as the Materials Project^3,6^. However, we note that these works do not use smearing as a variable for optimization (employing a fixed value instead), missing out on the benefits that smearing techniques may bring to the convergence of Brillouin zone (BZ) sampling, either by reducing the required computational cost for the calculations or improving their precision. In our work, we simultaneously consider *k*-point sampling and smearing temperature in the optimization process and combine these results with the guidelines from Prandini et al.^27^ to create reliable sets of parameters that are suited for different tradeoffs of precision and computational time. These parameters are then extensively tested for a wide distribution of materials.

In this work, we start by summarizing the general interplay between *k*-point sampling and smearing techniques targeted at improving the convergence and efficiency of DFT calculations. We then describe the automated workflows developed for benchmarking the choice of parameters and their effect on relevant physical properties. We identify distinct convergence behavior for various classes of materials and establish general criteria for which optimized parameters can be found. Finally, we discuss how these results are integrated in standard solid-state protocols, showcasing the different ways they are available for users through the open-source ecosystem of these codes.

## Results

### Smearing and *k*-point convergence

One of the main keys to numerical precision in all-electron and pseudopotential DFT calculations for crystals is *k*-point sampling. In this framework, errors arise due to the discretization of the BZ used to evaluate integrals of *k*-dependent functions, most notably the total energy^1,42^. Errors in total energies can also propagate to other quantities; e.g., forces on atoms and stresses. Numerical issues become even more evident for metallic systems, whose occupation function *f*(**k**) becomes discontinuous at the Fermi surface. Integration of these functions has notorious poor convergence with respect to a uniform sampling mesh^43^ since they are not differentiable. Practically, performing quantitatively accurate calculations for metals would, in this case, demand an exceedingly high number of *k*-points, making the calculations computationally unfeasible.

To solve this problem, various smearing techniques were developed to effectively smooth out the discontinuity of the electronic occupations at the Fermi level (see ref. ^44^ for a summary and review). By replacing the discontinuous occupation function with a smooth and differentiable one, smearing techniques are able to achieve exponential convergence of integrals with respect to the number of *k*-points. This process is often physically interpreted as adding a fictitious electronic temperature (*σ*) to the system^45^, since the broadening of the electronic occupations gives rise to a smeared density of states that can be written as the addition of an entropic term to the total free energy. By modifying *f*(*ϵ*) from the original physical one at *T* = 0K, the integrals converge faster, but the price to be paid is that they converge to a different value than the *σ* → 0 limit. More specifically, the smearing entropy that now enters the generalized free energy can be written as a power expansion in the smearing temperature^44^:

1 $$S=\mathop{\sum }\limits_{k=0}^{\infty }{c}_{k}{n}^{(k-1)}(\mu ){\sigma }^{k},$$

with coefficients *c*_*k*_ of the form:

$${c}_{k}={(-1)}^{k+1}\frac{1}{k!}{\int }_{-\infty }^{\infty }{\epsilon }^{k+1}\widetilde{\delta }(\epsilon )d\epsilon ,$$

where $\widetilde{\delta }$ is the broadening function employed.

Equation (1) leads us to a general observation that *S* depends not only on the smearing temperature *σ*, but also on the value of the derivatives of the density of states at the Fermi energy *n*^(*k*−1)^(*μ*). As further discussed, these two factors play an important role in driving the deviations from the *σ* = 0 total energy when introducing smearing.

**Fig. 1:**
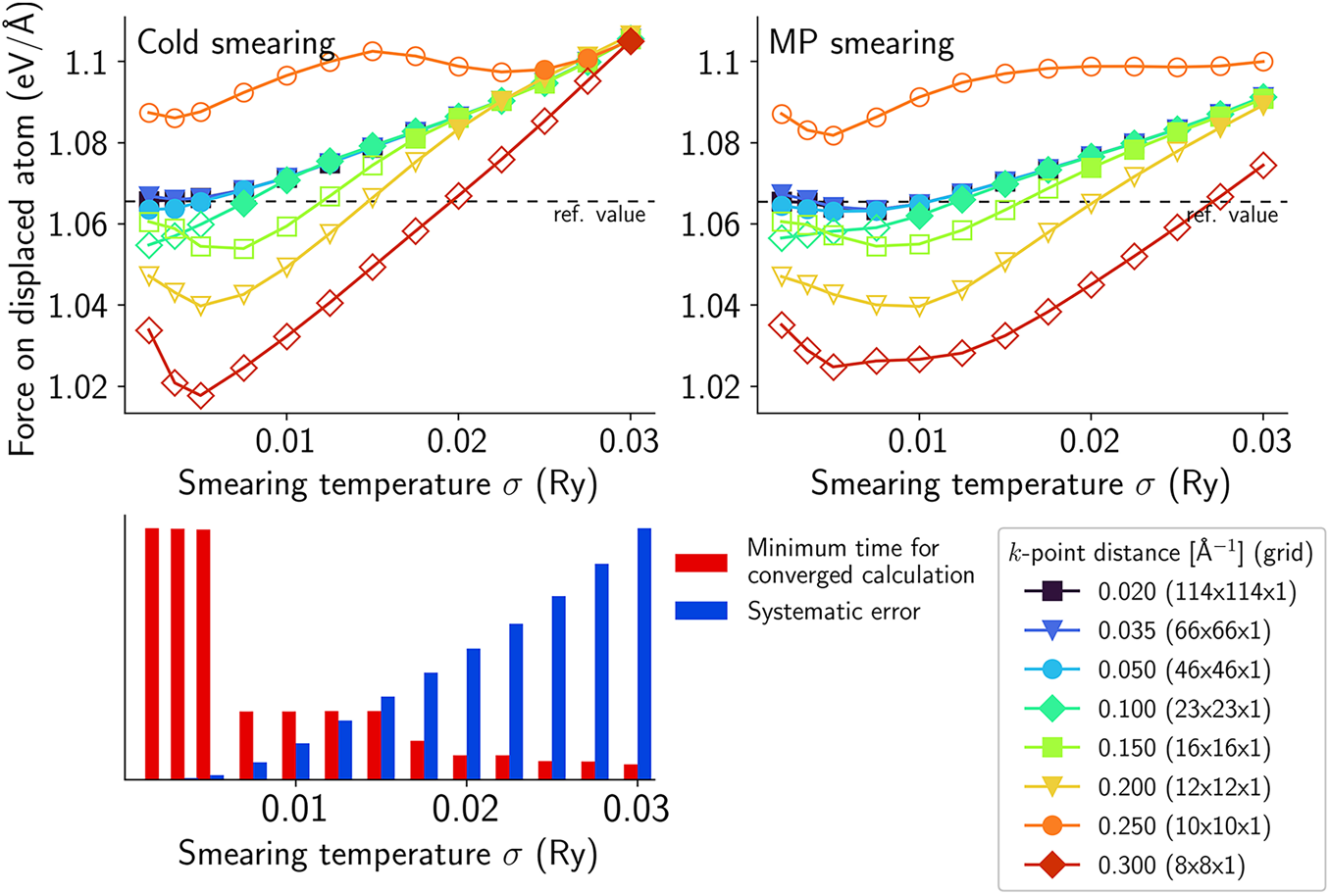
Convergence of forces as a function of *k*-point mesh and smearing for the metallic 1T phase of two-dimensional MoS_2_. Top: DFT-calculated force on an atom slightly displaced away from its equilibrium position as a function of smearing temperature, using Marzari–Vanderbilt cold smearing^46^ (left) and Methfessel–Paxton first-order smearing^43^ (right), for distinct *k*-point meshes. The horizontal dashed line indicates a reference value taken at the limit of the lowest smearing temperature (0.002 Ry), for which the 3 densest *k*-point meshes agree (up to 0.0035 eV/Å). Data points in the plot are shown as filled symbols if their calculated values match the results obtained using the densest k-point meshes within a tolerance of 0.0035 eV/Å. Empty symbols indicate that the results differ by more than this tolerance. Bottom: minimum amount of computational wall time required to converge a calculation w.r.t. *k*-points (red bars), for fixed threshold of 0.0035 eV/Å, and systematic error introduced (blue bars), both as a function of the smearing temperature *σ*, for the calculations done with cold smearing (upper left plot).

The practical interplay between *k*-point sampling and smearing temperature can be understood by tracking the value of a DFT-calculated property (e.g. total energy or forces) as a function of different combinations of parameters. This is done as an example in Fig. 1 for the metallic 1T phase of two-dimensional MoS_2_. In the upper plots in the figure, we show the calculated value of the force on an atom, slightly displaced out of its equilibrium position, as a function of the smearing temperature *σ* and *k*-point sampling for two smearing methods: Marzari–Vanderbilt cold smearing^46^ and first-order Methfessel–Paxton^43^ (see ref. ^44^ for a review of convergence behavior according to different smearing techniques). From these results, we observe distinct behavior in the two limits of *σ*. In the *σ* → 0 limit, the aforementioned problem of poor *k*-point sampling convergence becomes evident. Due to the fact that no variational principle exists for the convergence of the total energy (and other properties) with respect to *k*-sampling, results obtained by various coarse *k*-point grids are essentially random. That is, a small change in *k*-point sampling in this regime may lead to large changes in the calculated property. In this work, we understand that this source of errors due to finite sampling is dangerous and hard to quantify and should always be avoided; we call it the sampling (or statistical) error. In this low-smearing regime, satisfiable convergence is only achieved for extremely dense *k*-point grids (with mesh sizes approximately above 50 × 50 in the case of this 2D material), which is impractical for high-throughput workflows and other computational approaches.

On the other hand, we observe an enhanced *k*-point convergence as the smearing temperature increases. The higher *σ* is, the coarser the *k*-point meshes that can reproduce the results from the very dense *k*-sampling limit. This enables more computationally efficient calculations to be run while suppressing the source of errors that arise from poor *k*-point sampling. However, the problem with large *σ* is that higher entropic contributions kick in, so that the values for energies and other calculated properties start deviating from the true *σ* → 0 limit. This is observed in the upper plot in Fig. 1, where the curves for the different *k*-point meshes converge to the same values for high smearing, but deviate from the reference converged value *σ* → 0 (calculated with the densest *k*-point mesh and the smallest *σ*). In the following, we will refer to the errors that arise from smearing alone (i.e. when *k*-point sampling is properly converged) as systematic (smearing) errors.

The distinction between the aforementioned errors, namely systematic and sampling errors, is a central concept in this paper. Notably, these arise from different sources. While the former has a clear physical origin, stemming from entropic contributions associated with smeared occupations, the latter is stochastic and numerical, arising from the finite discretization used to sample the BZ. This also explains their different behavior. Systematic errors are quantifiable, smooth, and continuous functions of the smearing temperature (see Eq. (1)), even in those extreme cases where the derivatives of the density of states are very large (see Results section and discussion on materials with open *f*-shell elements). On the other hand, sampling errors are discontinuous and sharply varying, especially in the low *σ* regime (see Fig. 1, where the highest jumps in calculated properties arise from changing the k-point mesh for low values of *σ*). This distinction will be exploited in this paper to maximize the stability and reliability of the calculations for a fixed acceptable total error.

The optimized choice of *k*-point sampling and smearing parameters must be such that the interplay between systematic and sampling errors is kept under control. This “sweet spot", at which smearing effectively suppresses sampling errors but does not generate excessive systematic errors, is naturally dependent on the compromises between precision and computational cost one is willing to make. An effective control of errors should therefore provide a reliable way of setting this tradeoff, making sure that increments in computational cost are effectively converted into precision and vice-versa. In this work, we use smearing as a reliable parameter for suppressing the statistical errors introduced by *k*-point sampling, while using systematic errors as a controllable variable to adjust the tradeoff between precision and computational cost. The generalization of convergence studies as the one shown in Fig. 1 to a wide and representative range of materials gives rise to our defined SSSPr protocols. They form the set of statistically relevant parameters optimized for different precision vs. cost tradeoffs, while also being able to account for relevant differences in electronic structure across the materials landscape and thus providing an efficient framework for high-throughput workflows.

Finally, we note that our approach for optimizing these parameters relies on the assumption that errors from BZ sampling and smearing can be decoupled from other numerical approximations in DFT, such as the choice of basis sets and pseudopotentials. This assumption is justified, for instance, by the results of the benchmark between DFT codes by Bosoni et al.^47^. In this paper, the authors recommend using the very same smearing and *k*-point sampling schemes when comparing results between different DFT codes and show that, under such condition, two independent all-electron implementations yield a remarkable agreement on structural and mechanical properties over a broad range of materials, and nine other implementations based on pseudopotentials (but using different basis sets) also agree with those results. This supports the understanding that, if all other parameters are properly converged to obtain the correct DFT results for a given level of theory (e.g., choice of functional), then all codes should produce the same electronic band structure. Since the total energy functional can be written^1,42,48^ in a form such that the leading term of the energy is a sum over the band-structure eigenvalues ${\sum }_{i}{\epsilon }_{i}\equiv tr({\widehat{T}}_{s}+{\widehat{v}}_{KS})$, which is invariant under unitary changes of basis, the leading contribution to the system’s energy is a universal property, intrinsic to the material and independent of basis-set choice (but it depends on the choice of k-point sampling and smearing used to approximate the previous expression). This thus justifies the decoupling of BZ-sampling parameters from other numerical approximations in DFT, reinforcing the idea that the convergence analysis methods employed in this work are applicable to other codes and settings. A more precise discussion of the conditions under which methods and numerical values are transferable is discussed in more detail later.

### The benchmarking workflows

The optimization of parameters relies on data from DFT calculations from a wide variety of materials and combinations of parameters. As discussed in the Methods section, 269 structures were sampled from the Materials Cloud databases^4,49–51^ of three-dimensional (MC3D) and two-dimensional (MC2D) structures and used as starting point of the workflows discussed in this work. For each structure, an atom in the unit cell is randomly selected and displaced towards its nearest neighbor by 5% of the corresponding bond length. This avoids high-symmetry configurations where forces vanish by symmetry. Next, various DFT self-consistent calculations are performed with different combinations of *k*-point sampling and smearing parameters. We define a variable *λ* to represent the sampling of the BZ in each calculation. With units of 1/Å, *λ* encodes the maximum distance in reciprocal space, along each of the three directions defined by the reciprocal lattice vectors, between adjacent *k*-points of a uniform *Γ*-centered *k*-point mesh used in the calculation.

For smearing, Marzari–Vanderbilt cold smearing^46^ is chosen for all calculations, with *σ* ranging from 0.002 to 0.03 Ry. For insulators, we also perform calculations with fixed occupations and no smearing broadening, that can be used as *σ* = 0 reference value in this case. The choice of the cold smearing method is based on the fact that this broadening function is devised to yield total free energies independent of the smearing temperature up to and including the second order (from the entropy expansion in Eq. (1)), excluding the need of *a posteriori* corrections that are otherwise needed, for instance, in the Fermi-Dirac and Gaussian smearing techniques^44^. Furthermore, $\widetilde{\delta }$ in this approach produces a positive-definite occupation function, unlike the approach from Methfessel and Paxton^43^, where occupations can be unphysically negative. We note, however, that the cold smearing method has a nonmonotonic occupation function, as does Methfessel–Paxton, which in the case of insulators and semiconductors can lead to multiple mathematically valid Fermi energies and, if incorrectly identified, to spurious occupations and forces. This limitation was recently addressed by Dos Santos and Marzari^44^, who introduced a robust numerical protocol for Fermi-energy determination that removes this ambiguity. Their implementation, currently available in Quantum ESPRESSO, ensures that cold smearing remains reliable also in automated or high-throughput settings where the metallic or insulating character of a system may not be known a priori.

Nonetheless, we note that the same approach for benchmarking DFT quantities and statistical considerations used in the analysis of errors for the creation of protocols in this work are compatible with the other aforementioned smearing methods, readily available in multiple DFT codes, although the numerical values for optimized parameters may differ. For instance, we expect that repeating this benchmark for Gaussian smearing will result in smaller optimized values for the smearing temperature, given its quadratic depedence of the free energy with respect to *σ*, while requiring finer *k*-point meshes for equivalent precision (optimized values for *σ* using the Fermi-Dirac distribution can be obtained by scaling the *σ* parameters for Gaussian smearing down by a factor of $\lambda =\sqrt{2/3}\pi$, as pointed out in ref. ^44^). On the other hand, the numerical values obtained by our benchmarks using cold smearing can be used as a first approximation for resonable calcuations with other smearing techniques with similar *σ* dependence of the free-energy. This is the case of first-order Methfessel–Paxton. As illustrated in Fig. 1, the dependence of DFT energies and forces as a function of *σ* in the range considered here is in most cases similar enough between the two smearing techniques, suggesting that converged parameters for cold smearing are likely to be appropriate choices also for Methfessel–Paxton smearing.

As shown in Supplementary Table S1, this process generates 91 distinct calculations for each metallic structure and 28 for each insulating one, that can be labeled by the corresponding pair (*λ*, *σ*). The workflow automatically checks for electronic convergence (achieved in all cases) and keeps track of the final results for total energies (*E*), the force on the displaced atom (*F*), and pseudo stresses *Σ*, defined as the average of the diagonal components *σ*_*ii*_ of the stress tensor, where *i* = *x*, *y*, *z* for 3D materials and *i* = *x*, *y* for 2D (we use *Σ* in this case, given the customary use of *σ* in this context for smearing temperature).

### Analysis of errors

For each structure, a specific set of parameters must be chosen as the reference point to compare all other calculations. Ideally, this reference should be taken in the limit *λ* → 0 and *σ* → 0, i.e., an infinitely dense *k*-point mesh and zero smearing temperature, which is however not practically attainable. Therefore, for *k*-point sampling, we always take as a reference the densest mesh *λ*_ref_ = 0.035 Å^−1^. This value of *λ* provides *k*-point meshes with, on average, 202 and 888 irreducible *k*-points per atom in the primitive cells for 2D and 3D materials, respectively. The choice *σ*_ref_ for each material should instead guarantee that results are properly converged with respect to *k*-points. Therefore, *σ*_ref_ is chosen for each structure as the lowest value of *σ* for which the calculations with the three densest *k*-point meshes (*λ* = 0.035, 0.05, 0.10 Å^−1^) have total energies, forces, and (pseudo) stresses converged on *k*-points below given tolerances ${\tau }_{P}^{k}$, with *P* ∈ {*E*, *F*, *Σ*}. This means that sampling errors introduced on total energies, forces, and stresses, defined respectively as ${\varepsilon }_{E}^{k}$, ${\varepsilon }_{F}^{k}$, and ${\varepsilon }_{\Sigma }^{k}$, are such that:

2 $${\varepsilon }_{P}^{k}\le {\tau }_{P}^{k},\,\mathrm{where}\,{\varepsilon }_{P}^{k}(\lambda ,\sigma )\equiv | {P}_{(\lambda ,\sigma )}-{P}_{({\lambda }_{\mathrm{ref}},\sigma )}| ,$$

where *P*_(*λ*, *σ*)_ is the numerical value of the property *P* ∈ {*E*, *F*, *Σ*} computed in the DFT calculation having (*λ*, *σ*) parameters.

These ${\tau }_{P}^{k}$ thresholds represent maximum limits for the tolerable amount of sampling errors in a calculation and should be analyzed separately from the systematic errors introduced by smearing, ${\varepsilon }_{P}^{s}$. The latter is defined as the difference in the property value computed between the calculation with smearing *σ* and *k*-point distance *λ*_ref_ and the reference calculation with (*λ*_ref_, *σ*_ref_):

3 $${\varepsilon }_{P}^{s}(\sigma )\equiv | {P}_{({\lambda }_{{\rm{r}}{\rm{e}}{\rm{f}}},\sigma )}-{P}_{({\lambda }_{\mathrm{ref}},{\sigma }_{\mathrm{ref}})}| .$$

In this sense, sampling errors and systematic errors, ${\varepsilon }_{P}^{k}(\lambda ,\sigma )$ and ${\varepsilon }_{P}^{s}(\sigma )$ respectively, are calculated differently, as the former considers comparisons within the same smearing value and different *k*-point meshes, while the latter between the same (reference) *k*-point mesh and different smearing temperatures. The sum of the two errors is not simply the total error in a calculation (*λ*, *σ*), but is an upper bound to it:

4 $${\varepsilon }_{P}^{\mathrm{Total}}(\lambda ,\sigma )\le {\varepsilon }_{P}^{k}(\lambda ,\sigma )+{\varepsilon }_{P}^{s}(\sigma ),$$

as both errors can be either positive or negative.

Analyzing these two sources of errors separately, thus, eliminates misleading cases where sampling and systematic errors cancel out by chance. Additionally, defining numerical values for the convergence thresholds ${\tau }_{P}^{k,s}$ a priori is an ill-posed task, as one is ultimately interested in their propagating effect on errors of measurable properties of materials. Establishing a robust heuristic for determining these thresholds and using them to reliably control the precision vs. cost tradeoffs in the calculations is one of the purposes of the analysis that follows.

### General convergence trends

**Fig. 2:**
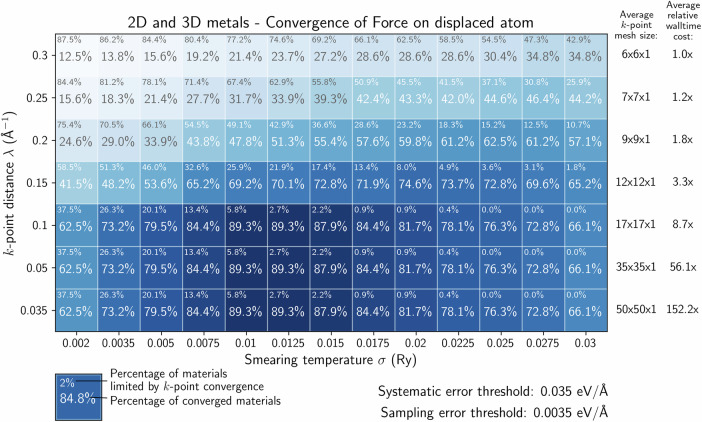
Force convergence for all 2D and 3D metals as a function of the *k*-points distance and smearing temperature. “Convergence", in this case, is considered reached by calculations producing both systematic and sampling errors below the thresholds ${\tau }_{F}^{s}$ and ${\tau }_{F}^{k}$, respectively. We use ${\tau }_{F}^{s}=0.035$ eV/Å and ${\tau }_{F}^{k}=0.0035$ eV/Å for this plot, noting that similar qualitative results are obtained with different threshold values. A visual legend is presented for an additional guide on the other metrics displayed in this plot. The metric on the relative wall time cost is defined as the ratio between the wall time required to run calculations for all materials with the respective *k*-point distance parameters and the wall time of the corresponding calculations with the computationally least expensive parameters in the analysis (least dense *k*-point meshes).

A first observation of the convergence trends for the calculated structures is shown in Fig. 2, for the specific case of metals, where we plot the percentage of materials whose calculated forces are converged as a function of the calculation parameters. Similar plots for errors in total energies and stresses have qualitatively similar characteristics as the one shown for forces. Here, both error contributions arising from *k*-points convergence and smearing are taken into account, such that a calculation is considered “converged" if and only if ${\varepsilon }_{F}^{k}\le {\tau }_{F}^{k}$ and ${\varepsilon }_{F}^{s}\le {\tau }_{F}^{s}$. Although the values for *τ*_*F*_ are still free parameters in the analysis, qualitative conclusions regarding the convergence behavior of the calculations can be drawn. First, the poor convergence at very low *σ* values can be observed on the left part of Fig. 2, even at very dense *k*-point meshes. Indeed, we can precisely quantify the percentage of materials that are bounded by *k*-point errors (as opposed to being dominated by systematic errors) by taking the limit of ${\tau }_{F}^{s}\to \infty$ at a fixed ${\tau }_{F}^{k}$. This is plotted at the upper left corner of each square in the heatmap of Fig. 2. We can see that calculations for low values of *σ* are mostly not converged due to sampling errors, and this metric monotonically decreases with *σ*, i.e., increasing the smearing temperature effectively enhances *k*-point convergence for all materials, as expected. However, total convergence also decreases on the right extreme of Fig. 2. In this regime, instead, the errors in the calculations are driven by the systematic errors introduced by the high smearing temperature. This highlights that an intermediate value of *σ* exists that minimizes both sampling and systematic errors for a fixed *k*-point mesh.

Furthermore, we can also note another trend when following the convergence vertically across *k*-point meshes in Fig. 2. As the density of *k*-points decreases (*λ* increases), the calculations become less computationally demanding, marked by the decrease in the relative wall time cost (numbers at the bottom of each square). Total convergence also decreases, but importantly we observe that the “sweet spot" in smearing also shifts to the right as *λ* increases. This produces a diagonal trend on the heatmap of Fig. 2, highlighting the fact that the “coarser" (i.e. less dense) a *k*-point mesh is, the more it benefits from a higher smearing temperature. This concept is at the core the optimization in protocols for high-throughput calculations: leveraging smearing temperature to enable more computationally efficient calculations with robust control of errors.

### Materials with open *f*-shell elements

**Fig. 3:**
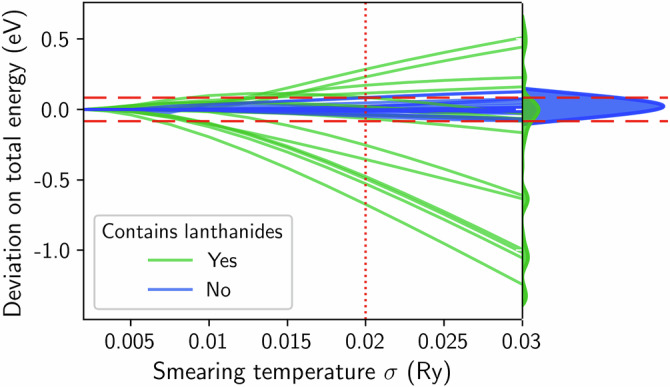
Systematic error on total energies as a function of smearing temperature. Materials with lanthanides are indicated by green lines and are those most sensitive to systematic errors. The curves on the right represent the distribution of errors at *σ* = 0.3 Ry for each class of materials (values interpolated from a discrete histogram). Horizontal red dashed lines indicate the error margins within which 85% of materials lie (±0.082 eV), measured at an intermediate smearing value of 0.2 Ry (dotted vertical line). Only calculations with the densest *k*-point meshes are considered, known to be converged with respect to *k*-points.

From our calculations, metallic materials containing lanthanide elements form the group of compounds most strongly affected by errors in total energies, arising mainly from the smearing temperature. This is illustrated in Fig. 3, where we plot the deviation on the total energy as a function of *σ* for each material. In the limit of high *σ*, materials containing lanthanides have remarkably higher systematic errors than the rest of the materials that do not contain open *f*-shell elements. These errors stem from the electronic structure of these materials, where valence *f*-states, due to their high localization in real space, generate low *k*-dispersive states in the system band structures, contributing to sharp peaks in the density of states. If these bands are close to the Fermi energy, these peaks lead to high derivative values *n*^(*k*−1)^(*μ*), which in turn lead to larger *S* with increasing *σ* (see Eq. (1)), resulting in high deviations of total energies observed in Fig. 3. In fact, the pathological case of lanthanide materials with smeared occupations was also observed by Bosoni et al.^47^, relating the strong deviation of the total free energy with smearing temperature with the presence of narrow *f*-bands close to the Fermi level. We also generate evidence for this hypothesis by calculating the projected density of states (PDOS) for all lanthanide-containing materials from Fig. 3, see Supplementary Fig. S1. All lanthanide materials that have large systematic errors have indeed their electronic DOS near the Fermi level dominated by sharp peaks arising from the lanthanide *f*-states. Therefore, structures containing lanthanide or actinide elements form a class of materials for which protocols should take special care due to their higher sensitivity to systematic errors introduced by increasing *σ*.

### Multiobjective optimization of precision and wall time

**Fig. 4:**
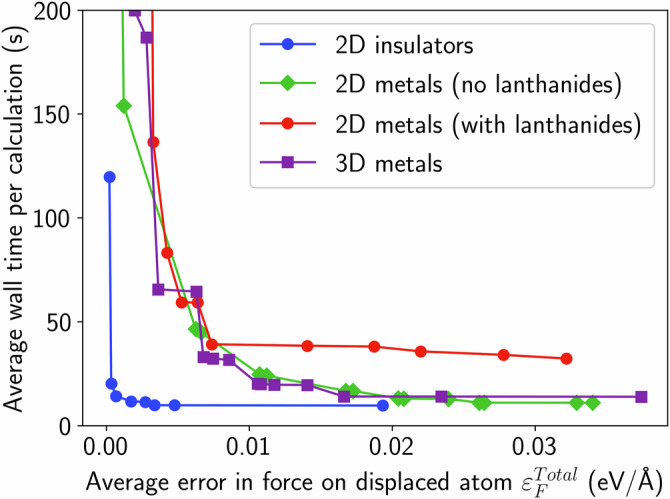
Pareto front between average wall time per calculation vs. average total errors on forces on the displaced atom (${\varepsilon }_{F}^{Total}$). Curves for the different material classes considered in this work are shown. Materials with lanthanides are defined as having at least one lanthanide element in their composition. All 3D metals considered here do not contain lanthanides.

Only a subset of all parameter combinations tested in our benchmarks minimize computational wall time for a fixed amount of average errors. This Pareto front, illustrated in Fig. 4, represents a collection of parameters that are most effective in converting increased computational resources into precision and vice-versa. Comparing the curves for 2D insulators and 2D metals, the faster convergence behavior of insulators is clear. As expected, these systems do not have the discontinuity problem at the Fermi level as metals do and, therefore, require less dense *k*-point meshes to achieve the same level of errors on calculated forces. This highlights opportunities for protocols in saving resources for calculations in systems known to be insulating. On the other hand, the curve of 2D metals including lanthanides shows that the higher systematic errors introduced by smearing for materials with open *f*-shells impose the need for higher computational resources to achieve the same level of precision. Furthermore, by comparing 2D and 3D metals, we do not observe a considerable difference in behavior with respect to the parameters that minimize the computational wall time for a given level of precision in the calculations. As such, in the following we group these two groups of materials into a single class of metals.

The analysis from Fig. 4, however, deals with sampling and systematic errors on equal footing. Nevertheless, as discussed earlier, using smearing temperature to suppress the less controllable sampling errors is beneficial, since it provides a more controllable source of errors according to desired precision × cost tradeoff. A reliable Pareto front for our optimized parameters, in this way, takes that into consideration by imposing additional constraints on the composition of the total error in the calculations. In this work, we impose the constraint that, within any calculation, tolerable sampling errors should be at most 5% of the value of systematic errors. This ensures that, at any level of precision required, smearing correctly guarantees the convergence of *k*-point sampling, preventing slight changes in the electronic structure or BZ sampling from generating uncontrollable errors. The rationale for this threshold is two-fold: (i) it reflects our experience with BZ sampling in high-throughput workflows^51^, and (ii) it is robust enough to yield stable optimized parameters across different tolerance factors in our benchmark (see Supplementary Fig. S2). This allows different codes to implement the same protocols by enforcing error tolerances, rather than directly matching smearing and k-point sampling parameters, in cases where the smearing scheme differs from the one employed here.

**Fig. 5:**
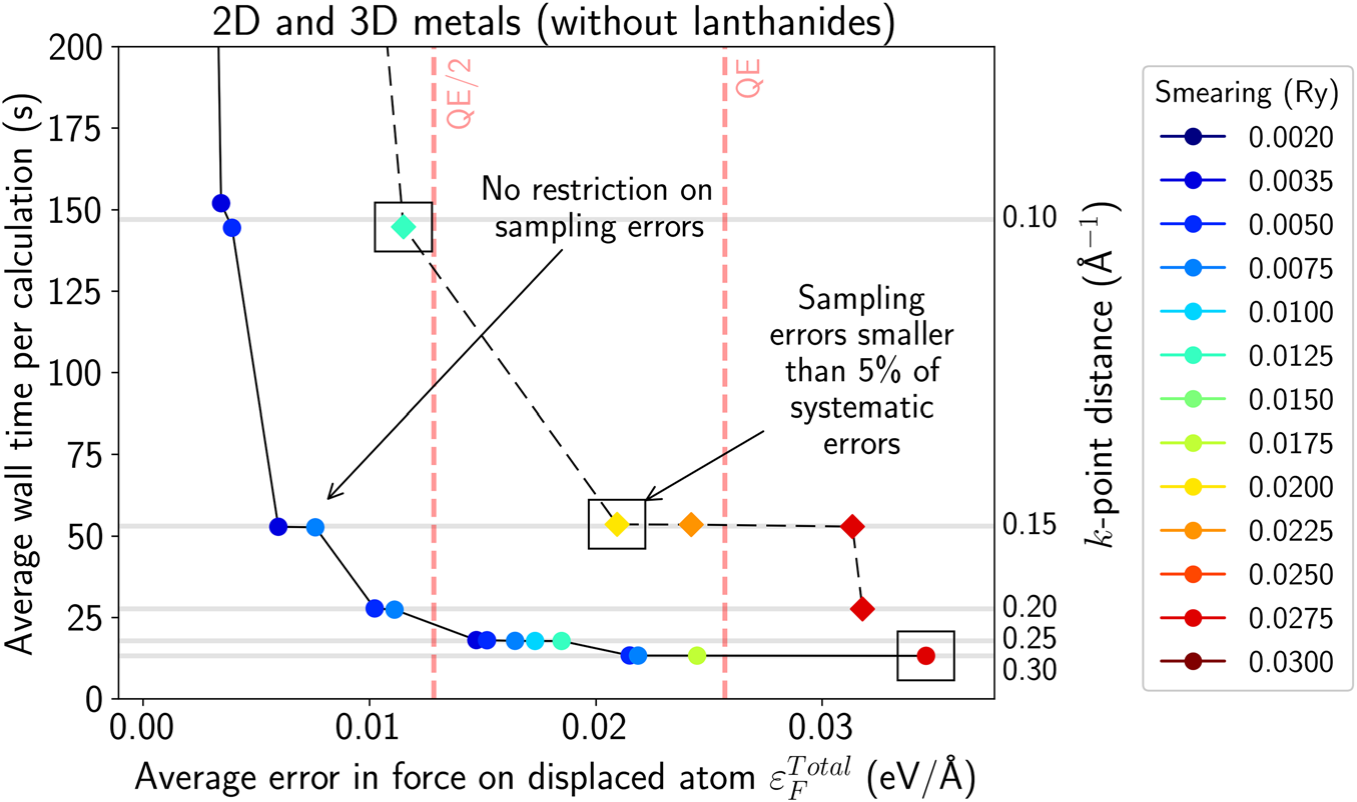
Optimized parameters on the Pareto front between precision and computational cost. The *x*-axis represents the average total error on the force on the displaced atom (${\varepsilon }_{F}^{Total}$) for all metals without lanthanides (2D and 3D). The *y*-axis represents the average computational wall time per calculation, which has a direct mapping to the *k*-point mesh size (axis on the right). The dashed black line represents the Pareto front with the additional constraint of sampling errors being smaller than 5% of the systematic errors (${\varepsilon }_{F}^{k} < 0.05{\varepsilon }_{F}^{s}$), while the continuous line is the unconstrained one. The vertical red dashed lines represent reference thresholds for forces as the stopping criteria in a DFT relaxation. The line on the right (QE) represents the default value of Quantum ESPRESSO of 10^−3^ Ry/bohr (0.026 eV/Å), while the one on the left (QE/2) represents a stricter reference of 5 ⋅ 10^−4^ Ry/bohr (0.013 eV/Å, i.e. half of the QE value). Optimized parameters are chosen on this Pareto front and indicated by black rectangles (see Discussion section). As each data point is colored according to the value of *σ*, the optimized parameters (*λ*, *σ*) can be directly read out of the plot.

Figure 5 illustrates the analysis of the Pareto front for metals with the additional constraint on error composition, compared to the unconstrained version shown in Fig. 4. By considering sampling and systematic errors distinctly, we observe that the additional constraint shifts the suggested *k*-point sampling values to denser meshes (i.e. smaller *λ*) in order to minimize sampling errors in favor of systematic ones. In doing so, the suggested values for the smearing temperature increase accordingly. Furthermore, in order to quantify the magnitude of errors, we can compare errors with the thresholds on forces used in relaxation calculations. Having forces on all atoms below a threshold is one of the criteria considered for stopping the ionic minimization and atomic position updates in relaxation calculations. Specifically, in Fig. 5, we show the default forces threshold employed in Quantum ESPRESSO, together with a stricter reference of half of this value.

An additional and important advantage of the analysis presented in Fig. 5 over the convergence heatmaps of Fig. 2 is its higher independence of the thresholds considered. First, at variance with the heatmap analysis, the Pareto front approach is totally free from a threshold on systematic errors (*τ*^*s*^), as the average error in the calculations is presented as an independent variable on the *x*-axis (as opposed to serving as a fixed cutoff value as in Fig. 2). The only adjustable threshold parameter, in this case, becomes *τ*^*k*^, as it is needed to define the lowest value of smearing producing reliable calculations for a material (*σ*_ref_), according to the definition of Eq. (2). However, we also observe that, in practice, the optimized parameters obtained from these analyses are independent of *τ*^*k*^ over a wide range of values, as illustrated in Supplementary Fig. S2. This can be attributed to the design choice of suppressing sampling errors in favor of systematic errors, contributing to an additional level of robustness in the analysis.

Finally, as illustrated in Supplementary Fig. S3, the optimized parameters that minimize the computational time and errors for energies or stresses are effectively the same as those for forces presented here. Therefore benchmarking of any of these three properties essentially leads to the same result in terms of protocol parameters.

## Discussion

Based on the previous results, we define the standard solid-state protocols (SSSPr) for plane-wave DFT calculations. As mentioned earlier, the name was chosen to mirror the standard solid-state pseudopotential libraries (SSSP)^27^, indicating their seamless interoperability and the fact that they are designed to be used together. Similar to ref. ^27^, our benchmarks were done using the Quantum ESPRESSO distribution, but users from other plane-wave pseudopotential codes (e.g. VASP^40,41^, Abinit^52^), as well as all-electron codes (e.g. Fleur^53^, WIEN2k^54^) that rely on smearing for facilitating convergence of BZ sampling, may also benefit from this analysis and results (see ref. ^47^ for a review of protocols across different codes). As mentioned before, the numerical values obtained with Marzari–Vanderbilt cold smearing in these protocols may be used as a first approximation for other smearing techniques with similar dependencies of the free energy with respect to *σ* (e.g. first-order Methfessel–Paxton). For smearing techniques different from the one used here (e.g., Gaussian broadening), the methodology developed in this work can be equally applied to optimize the corresponding numerical parameters.

The protocols are designed to represent three distinct choices according to different precision and efficiency tradeoffs, and are named Fast, Balanced and Stringent. The values of the optimized parameters for each protocol can be directly read from the optimized Pareto front from Fig. 5 (indicated by black rectangles) and are summarized in Table 1. To validate these numbers, we have repeated the analysis 1000 more times on randomly drawn subsamples of the data following the bootstrapping method (sampling with replacement). Supplementary Table S3 shows the results of the average parameters obtained by this analysis, which agree with the final values of the protocols within one standard deviation.

**Table 1:** Standard solid-state protocols and their respective optimized parameters

Protocol	*σ*_cold_ (Ry)	*σ*_cold_ (eV)	*λ* (Å^−1^)	Notes
Stringent	0.0125	0.1701	0.10	Always recommended for metals with lanthanides/actinides
Balanced	0.0200	0.2721	0.15	Always recommended for insulators
Fast	0.0275	0.3742	0.30	For testing purposes

*σ*_cold_ represents the numerical values of smearing temperature found through the optimization using Marzari-Vanderbilt-DeVita-Payne cold smearing^46^, expressed both in Ry and eV (we note that, in the input of Quantum ESPRESSO, the value is expressed in Ry). *λ* represents the optimized *k*-point distance for each choice of smearing, as defined in the *Benchmarking workflows* section in the Results. As discussed in the main text, the Balanced protocol is always the optimal choice for insulators. The Stringent protocol, on the other hand, is the default recommendation for metallic systems containing lanthanide or actinide elements.

In the following, we present a general overview of each protocol, along with their suggested SSSP pseudopotential family^27^. We note that these choices are made based on the results obtained for metallic materials not including lanthanides nor actinides, and we suggest ways for which they can be adapted for insulators and metals with lanthanides/actinides.

Fast: The fastest choice, meant for testing purposes or preliminary structure relaxations. It is not guaranteed to produce precise quantities with respect to the baseline reference, but it should still efficiently generate consistent and reproducible results. To be used alongside SSSP Efficiency pseudopotentials.

Balanced: On average, 3.6× slower for metals than the Fast protocol. Suitable for high-throughput screening and most practical DFT applications. For metals, it yields an average error on forces of 20.9 meV/Å and 4.4 meV/atom for total energies. The SSSP Efficiency pseudopotential family is the recommended choice in this case.

Stringent: 2.7× slower than Balanced (or an order of magnitude slower than Fast) on average. Produces average errors on forces of 11.5 meV/Å for 2D and 3D metals, and average errors on total energies of the order of 1.7 meV/atom. It is the highest precision choice before reaching the high slopes of the curve between required wall time × average errors. SSSP Precision pseudopotentials, along with their optimized plane-wave cutoff energies^27^ are suggested to be used here for maximum precision.

The optimized parameters for the protocols are chosen based on an analysis of metals without lanthanides/actinides. However, other classes of materials may have different needs for parameters that optimize precision and efficiency. More specifically, insulators suffer much less from sampling errors and allow coarser meshes to be used, decreasing the computational cost required. On the other hand, materials with lanthanide or actinide elements particularly benefit from smaller smearing values due to high systematic errors, as discussed in the Results section. In this work, however, it has been decided that each protocol should represent a single and unified choice of parameters across material classes. This design choice is aimed at the simplicity of how each protocol is defined and is especially useful when the metallic/insulating nature of the material is not known a priori. In this way, we aim to solve the aforementioned issue by the following procedure:

i. If the structure is insulating: The Balanced protocol is the most recommended choice since it already provides average errors on forces that are one order of magnitude smaller than for metals. Running Stringent, in this case, would imply a cost 1.82× higher and only minor improvements in errors.
ii. For metals including lanthanide/actinide elements: Employing the Stringent protocol is always encouraged in this case. The Balanced protocol smearing value of 0.02 Ry yields higher errors on forces that lie above the default thresholds of Quantum ESPRESSO.

**Fig. 6:**
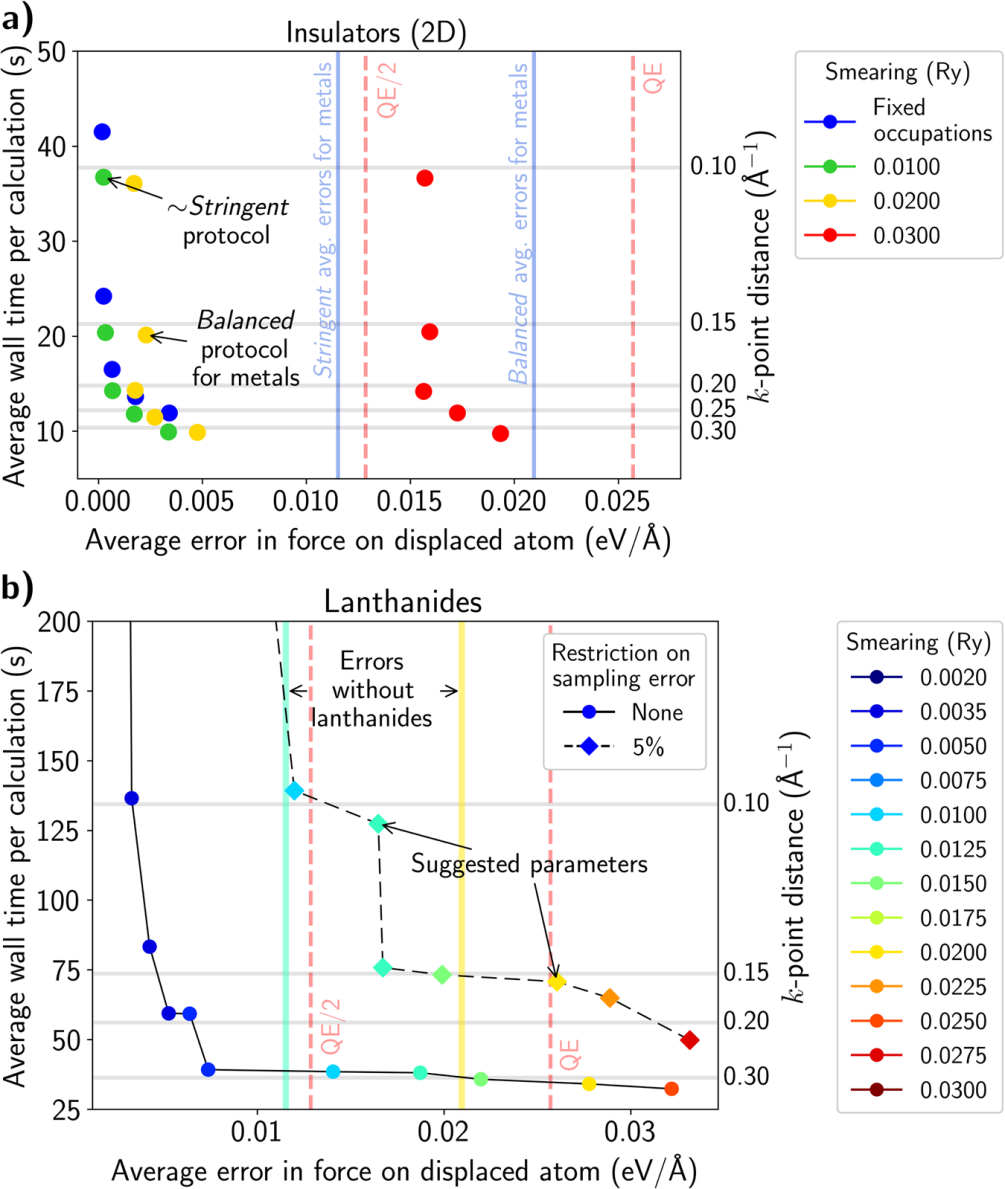
Average wall time per calculation versus the average errors on the force on a displaced atom for insulators (top) and metals with lanthanides (bottom). The parameters chosen for the protocols (Table 1) are indicated in the plots by arrows. Additional vertical bars are also used to indicate the reference of average errors produced by the protocols on simple metals (Fig. 5). The term ~Stringent protocol in Fig. **a** carries a ~symbol to indicate that it represents the parameters that are closest to the ones chosen for this protocol (0.0125 Ry instead of 0.01 Ry of smearing as calculated for insulators here). From the top panel, it is observed that the Stringent protocol is never needed for insulators, as the Balanced choice produces more efficient calculations with comparable errors. On the other hand, the bottom panel illustrates the need for the Stringent protocol for metals with lanthanides, in which the Balanced protocol produces excessive errors in these cases. Refer to Fig. 5 for further explanation of the plots.

These guidelines are justified in Fig. 6, where we show that running the Balanced protocol for insulators already produces satisfiable results with lower resource needs, but might be not enough for lanthanide materials, which can strongly benefit from the Stringent protocol. In cases where structures run in high-throughput workflows are unknown to be metallic or insulating, a standard practice can be to run non-lanthanide-containing structures with the Balanced protocol, then check their electronic structure and rerun it with the Stringent protocol for metallic cases, depending on the precision requirements.

As a further benchmark and validation of the choice of protocol parameters, we investigate, for a smaller subset of ~20% of the metallic materials, how errors on total energies, forces and stresses propagate to other material properties, namely the equilibrium lattice constant and phonon frequencies. From our design choice of protocols, we can safely assume that the largest part of sampling errors are suppressed. This enables us to use smearing temperature as the unique variable for measuring errors. In this way, we compute the relaxed structure and *Γ*-phonon optical frequencies using only the densest *k*-point mesh used in this work (see Supplementary Table S1) and track how these values evolve as a function of *σ*. Supplementary Fig. S4 shows the relative errors for relaxed lattice constants and optical phonon frequencies as a function of smearing temperature for the calculations of both 2D and 3D materials. From these results, we can estimate the average values of errors in these properties that our given choice of protocol parameters are expected to have, as displayed in Table 2.

**Table 2:** Estimate of average percentage errors for the Stringent and Balanced protocols

	Average percentage error			
	Lattice constants		Largest freq. at Γ	
Protocol	2D	3D	2D	3D
Stringent	0.04%	0.03%	0.26%	0.39%
Balanced	0.1%	0.05%	0.41%	0.76%

Estimated errors are reported for equilibrium lattice constants and for the phonon frequency at Γ of the highest (optical) branch.

Our protocols have been seamlessly integrated within the Quantum ESPRESSO environment and scaled to high-throughput calculations through the AiiDA framework, suiting different use cases. First, they are implemented within the Materials Cloud^55^ tool “Quantum ESPRESSO input generator". On the tool website, users can upload crystal structures in various formats and get input files generated automatically with meaningful input parameters chosen based on the benchmarks from this work, along with the SSSP pseudopotentials and related parameters extensively benchmarked by Prandini et al.^27^. This provides a useful starting point for calculations with new structures and is especially helpful for beginners with the code. For automating this procedure, users of any workflow for the Quantum ESPRESSO pw.x code in aiida-quantumespresso have access to these protocols and their parameters through the simple methods get_builder_from_protocol() available in each of the AiiDA WorkChain workflow classes^38^. This also makes it suitable for running high-throughput workflows. Furthermore, for users outside the python ecosystem, our protocols are available in the AiiDAlab Quantum ESPRESSO app^56^, which is an interactive platform with a GUI application where users can use ready-to-run workflows for materials properties and analyze results within a web browser. Being heavily automated and powered by AiiDA in the backend, these workflows can rely on our protocols as a part of setting up the required calculations on the fly.

In this way, this work identifies protocols for high-throughput DFT calculations by optimizing the precision × cost trade-off from the interplay between *k*-point sampling and smearing techniques. For a rigorous control of the sources of errors, we introduce an analysis that decouples numerical errors arising from smearing (systematic errors) and sampling errors due to finite *k*-point meshes. We then propose a criterion for suppressing the less controllable errors from *k*-sampling in favor of systematic errors, in a way that a reliable balance between numerical precision × computational cost can be achieved. By extensively benchmarking these parameters for a wide range of materials, we determine different choices of *k*-sampling and smearing parameters that best optimize the required computational cost for a given level of precision required. We then propose heuristics on how to apply those choices for different classes of materials, namely: metallic systems with lanthanide elements, in which systematic errors are chronically more severe, and insulators, that do not suffer from sampling errors as strongly.

From these results, we define the standard solid-state protocols (SSSPr), that are not only suitable for high-throughput workflows, but also for running new calculations for specific materials with minimal initial investigation. By automatically providing input parameters for any structure, these protocols act as abstractions, hiding layers of complexity of the code’s internal algorithms. This is beneficial for making the code both more robust and user-friendly, avoiding potential errors due to inadequate parameter selection. More generally, protocols can be an essential piece in building interoperable software suitable for robust computational workflows. They enable workflows to be treated as building blocks of larger workflows for materials design, suiting different needs and requirements.

Here, we specifically establish protocols for self-consistent DFT calculations in Quantum ESPRESSO by benchmarking the most fundamental quantities of the theory, i.e. total energies, forces, and stresses. In this way, our protocols provide the basic parameters related to BZ sampling, in addition to pseudopotentials and plane-wave cutoffs already provided by ref. ^27^. We note, though, that further tests may be required when running calculations targeted at other properties, where also additional parameters may need to be considered. For instance, in the computation of linear-response properties such as phonons, convergence of the **q**-grid of monochromatic perturbations is also needed. In these cases, we also note the emergence of advanced BZ integration methods using nonuniform grids^57^ as promising approaches. Finally, similar techniques as those developed in this work can be applied to create protocols for many other simulation codes beyond Quantum ESPRESSO, either by direct application of the numerical results of this work, when applicable, or by rerunning the benchmark, e.g. if the smearing or *k*-sampling stategies are different. We note that our methods of analysis are expected to be independent of the basis set used (plane waves, Gaussians, localized orbitals) or the all-electron vs. pseudopotential implementation (norm conserving, ultrasoft, projector-augmented waves) and thus broadly universal.

## Methods

The crystal structures in this work were taken from the Materials Cloud three-dimensional and two-dimensional crystals databases (MC3D and MC2D)^4,49,51,58^. These are open materials databases of computational DFT simulations of crystals originating from experimentally known compounds from MPDS^59^, COD^60^, and ICSD^61^. The compounds used in our calculations were randomly selected among the structures containing at most 14 atoms in the unit cell available in the databases. Supplementary Table S2 displays the number of structures selected for the DFT benchmarking workflows. The exact material structures used, along with their calculation results, are available in the Materials Cloud Archive enry associated to this work^62^ in the form of AiiDA archive files.

The computational workflows were developed and managed with AiiDA^21–23^, providing seamless scalability and provenance tracking of all the calculation results. The DFT calculations were run with Quantum ESPRESSO v7.0^35–37^, employing the standard solid-state pseudopotentials family SSSP PBEsol Efficiency 1.2^27^ and PBEsol as exchange-correlation functional^63^. Thanks to the AiiDA plugin for Quantum ESPRESSO^38^, inputs for calculations were automatically generated with the appropriate recommended pseudopotentials and the corresponding plane-wave energy cutoffs, as extensively benchmarked by Prandini et al.^27^. Furthermore, a stricter threshold of 0.2 ⋅ 10^−9^ Ry/atom for the self-consistent field (SCF) electronic minimization was employed. As the average number of atoms per structure was 5.2, this renders an average SCF threshold of ~10^−9^ Ry, being three orders of magnitude smaller than the one employed by default in Quantum ESPRESSO (10^−6^ Ry). The same parameters were employed for the relaxation calculations, using thresholds for ionic minimization of 0.5 ⋅ 10^−5^ Ry/atom for total energies, 0.5 ⋅ 10^−5^ Ry/*a*_0_ for forces and 0.1 kbar for pressure. *Γ*-phonon frequencies were calculated from these geometries through density-functional perturbation theory (DFPT), as implemented in the ph.x module from Quantum ESPRESSO. One assumption of this work is that the optimized parameters obtained from the analysis of these results are transferable to other calculations with comparable settings, i.e., other GGA exchange-correlation functionals (e.g., PBE) and pseudopotential families (e.g., SSSP Precision^27^).

## Supplementary information

Supplementary Information

## Data Availability

Full provenance of the high-throughput DFT calculations in this work is generated through AiiDA and available in the Materials Cloud Archive^62^, along with a Jupyter Notebook for generating interactive plots of Fig. 2 with variable error thresholds.

## Code availability

The input generator for Quantum ESPRESSO is available at https://qeinputgenerator.materialscloud.io. The high-throughput compatible implementation of the protocols is available in the AiiDA plugin for Quantum ESPRESSO (source code at https://github.com/aiidateam/aiida-quantumespresso).
