# Supplementary material for: Accurate and efficient protocols for high-throughput first-principles materials simulations
Source: NPJ Comput Mater. 2026 Apr 29;12(1):272. doi: 10.1038/s41524-026-02097-8 (PMC13473013; doi:10.1038/s41524-026-02097-8)
Supplement: Supplementary file 1 [file 41524_2026_2097_MOESM1_ESM.pdf]

Supplementary Information: Accurate and efficient protocols for high-throughput first-principles materials simulations

Gabriel M. Nascimento,[1,2,3] Flaviano José dos Santos,[2,3] Marnik Bercx,[2,3] Davide Grassano,[1] Giovanni Pizzi,[2,3] and Nicola Marzari[1,2,3]

[1] *Theory and Simulation of Materials (THEOS), École Polytechnique Fédérale de Lausanne, Lausanne 1015, Switzerland*

[2] *PSI Center for Scientific Computing, Theory and Data, 5232 Villigen PSI, Switzerland*

[3] *National Centre for Computational Design and Discovery of Novel Materials (MARVEL), Paul Scherrer Institute PSI, 5232 Villigen PSI, Switzerland*

I. SUPPLEMENTARY FIGURES

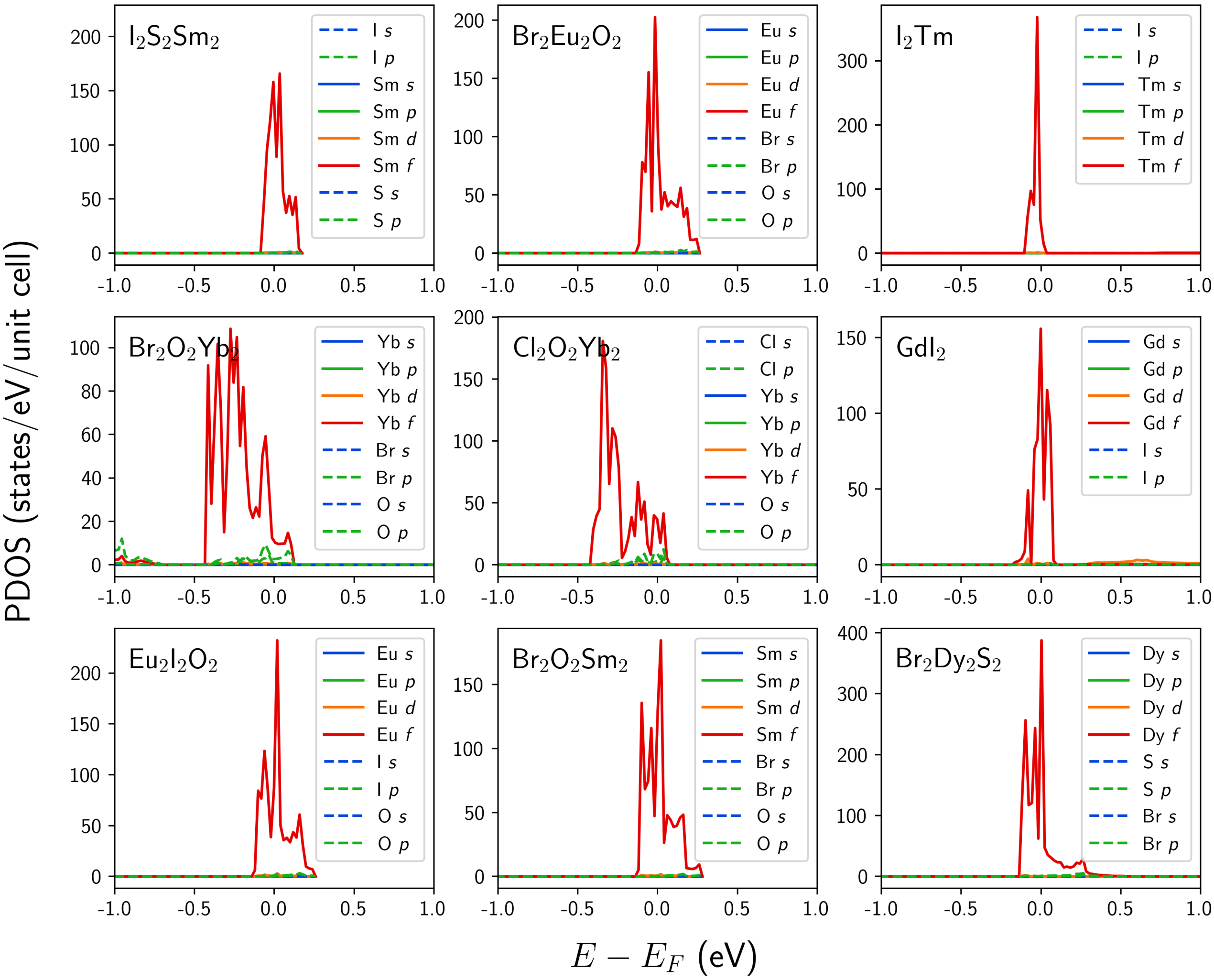

Supplementary Figure S1. Projected density of states (PDOS) of materials containing lanthanides with the highest systematic errors observed in Fig. 3 of the main paper. In every case, the presence of a high number of f-states introduced by the lanthanide element (red lines) around the Fermi level generates sharp peaks in the density of states of these materials.

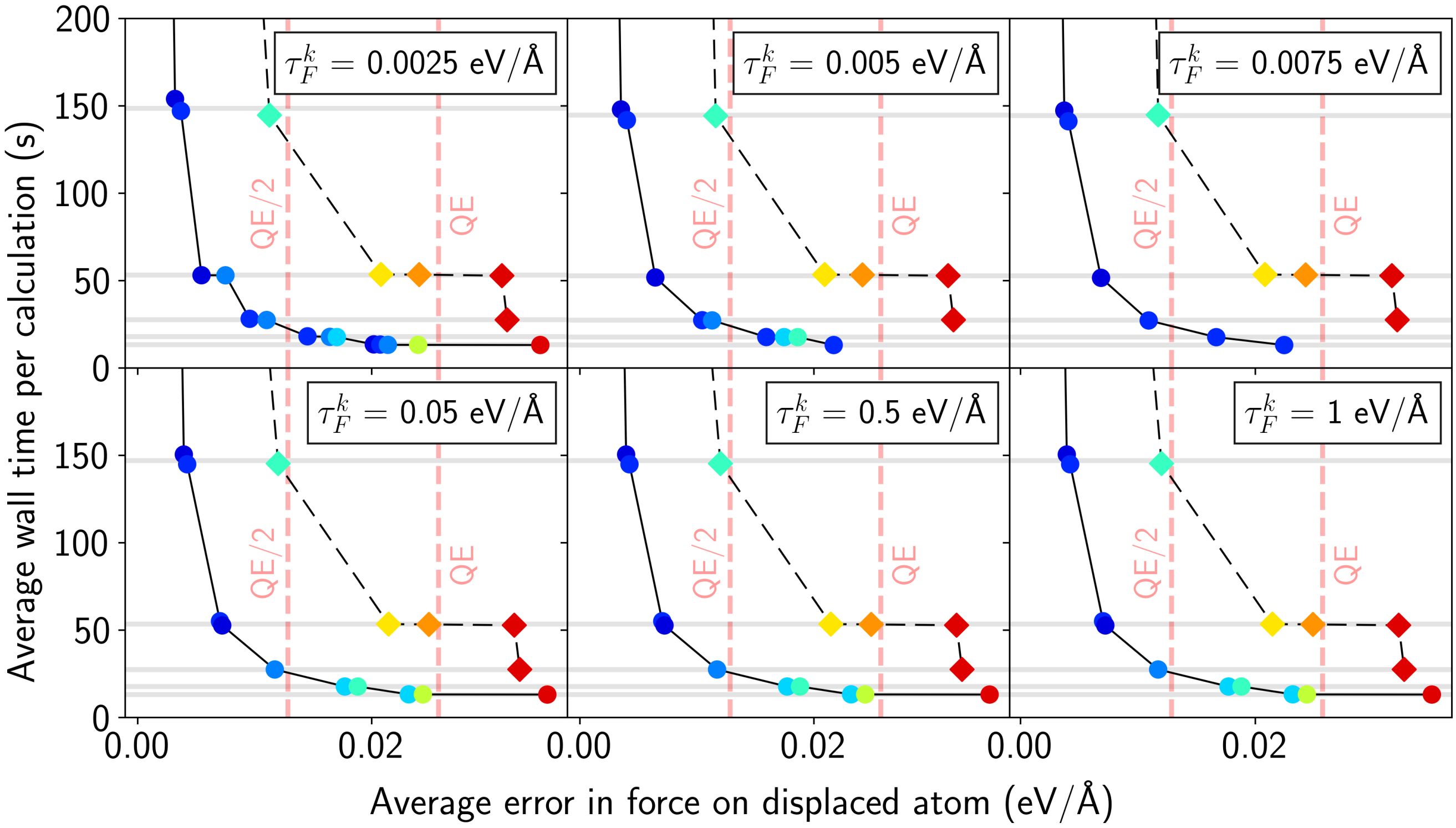

Supplementary Figure S2. Pareto front analysis as presented in Figure 5 of the main paper, for different values of the threshold on sampling errors (τ^k) used to determine σ_{ref} of each material. As in Fig. 5, the dashed lines represent the Pareto front with the additional constraint on the composition of the errors and are independent of the value of τ^k in this range, as opposed to the unconstrained version (continuous lines).

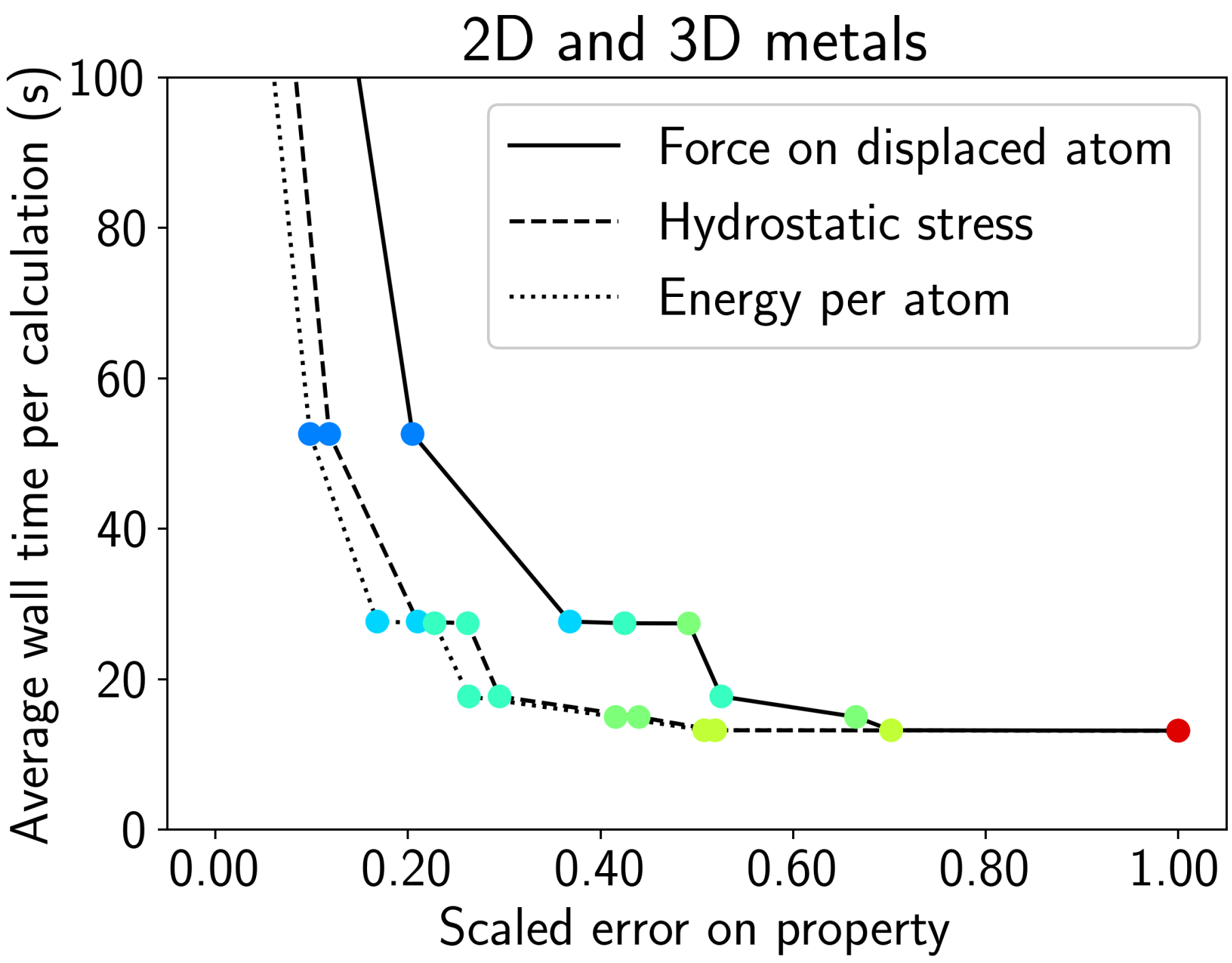

Supplementary Figure S3. Pareto front for all three properties tracked in the benchmarking calculations. The y-axis displays the average computational wall time per calculation, while the x-axis represents a min-max normalized error on total energies, forces, and stresses. More precisely, as each combination of parameters (λ, σ) produces an average total error on property P of $\bar{\varepsilon}_P(\lambda, \sigma)$, the values plotted on the x-axis are defined to be $x = [\bar{\varepsilon}_P - \min(\bar{\varepsilon}_P)]/[\max(\bar{\varepsilon}_P) - \min(\bar{\varepsilon}_P)]$, where $\min(\bar{\varepsilon}_P)$ and $\max(\bar{\varepsilon}_P)$ are the minimum and maximum value of $\bar{\varepsilon}_P$ over all possible (λ, σ) combinations calculated in this work. As in other plots, each point represents a pair of parameters and is colored according to the value of σ. As mentioned before, the y coordinate of each point has a direct mapping to its λ value, such that the optimized parameters for each property can be directly inferred and are essentially the same.

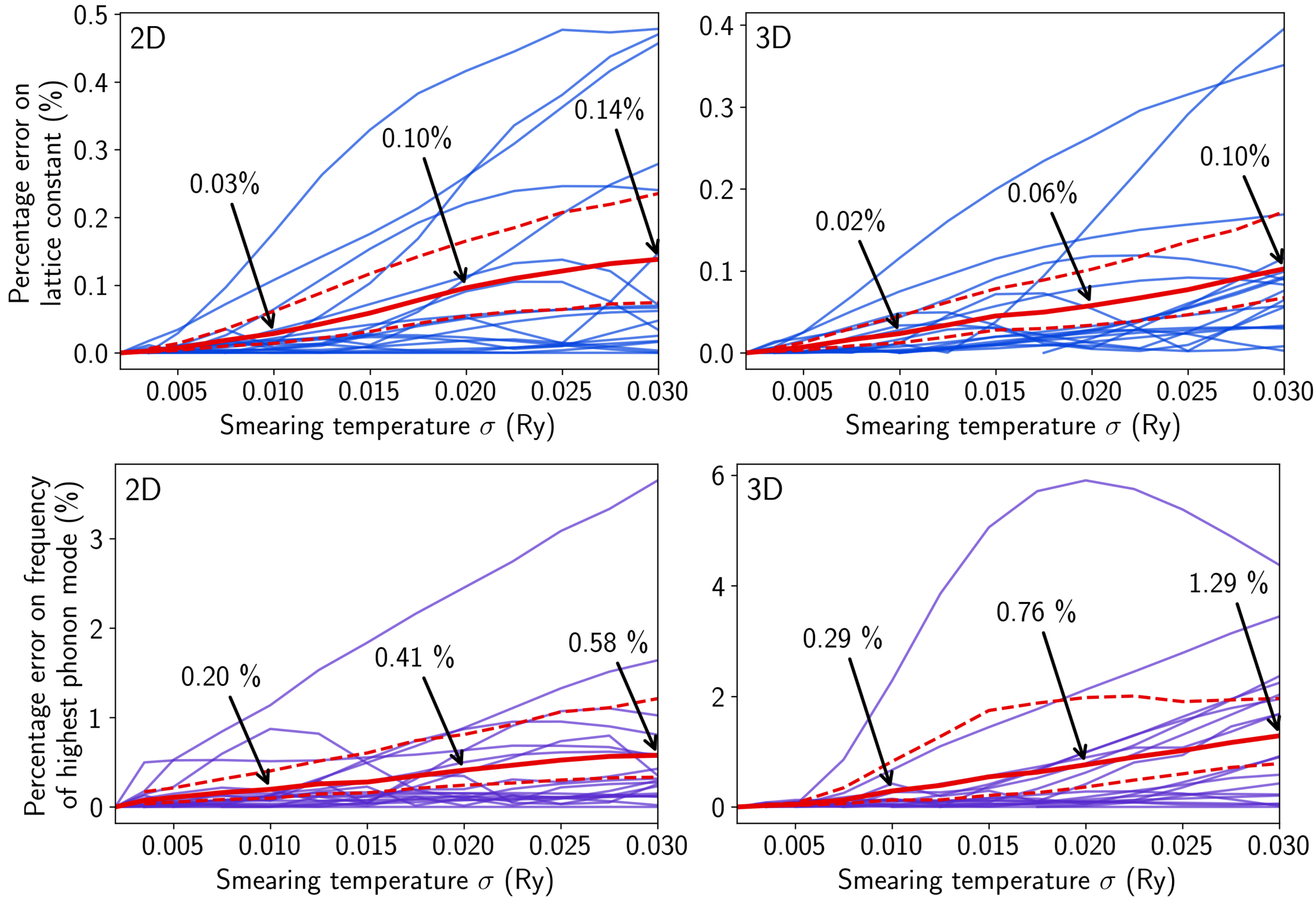

Supplementary Figure S4. Relative errors on "higher-order" properties as a function of smearing. Plots on the top: percentage errors on relaxed lattice constant for 2D (left) and 3D (right) metals. Plots on the bottom: percentage errors calculated phonon frequency of the highest-energy optical phonon mode at Γ. Continuous red lines represent averaged error values, with black arrows displaying their numerical values at $\sigma = 0.1, 0.2, 0.3$ Ry. Dashed lines indicate estimates for the 95% confidence intervals of the average error, calculated through bootstrapping. In all cases, calculations are carried out using the densest k-point meshes, and the smearing reference is taken according to σ_{ref} defined for each material (see section on analysis of errors in the main paper).

II. SUPPLEMENTARY TABLES

	Smearing temperature σ (Ry)	k-point distance λ (1/Å)
Metals	0.002, 0.0035, (0.005 - 0.030, 11)	0.035, (0.05 - 0.30, 6)
Insulators	0.0*, 0.01, 0.02, 0.03	

Supplementary Table S1. Smearing temperature values (σ) and k-point sampling distances (λ) employed in the benchmarking of DFT calculations in the workflows. The notation (x - y, n) represents a equally spaced sequence of n values within x and y, both included. The value 0.0* represents DFT calculations run with smearing disabled (fixed occupations).

Class		# structures
2D	insulators	45
	metals without lanthanides	59
	metals with lanthanides	20
3D	metals	145

Supplementary Table S2. Number of structures selected for the DFT benchmarks, separated by material class.

Protocol	λ (Å^{-1})	σ_{cold} (Ry)	$\tilde{\sigma}$ (eV)
Stringent	0.10	0.0125	0.0136 ± 0.0013
Balanced	0.15	0.0200	0.0211 ± 0.0013

Supplementary Table S3. **Optimized parameters from bootstrapping**. The Pareto front from Fig. 5 is recalculated 1000 other times by randomly resampling materials from the total number of metallic systems available. The sample size is the same as the total number of materials (sampling with replacement). For the Stringent and Balanced protocols, $\tilde{\sigma}$ represents the average value and standard deviation of the optimized smearing temperature across all 1000 resampling runs. This is not done to the Fast protocol, as its parameters are not obtained through this optimization method.
